# Preparation and Characterization of Starch/Empty Fruit Bunch-Based Bioplastic Composites Reinforced with Epoxidized Oils

**DOI:** 10.3390/polym13010094

**Published:** 2020-12-29

**Authors:** Jianlei Yang, Yern Chee Ching, Cheng Hock Chuah, Nai-Shang Liou

**Affiliations:** 1 Department of Chemical Engineering, Faculty of Engineering, University of Malaya, Kuala Lumpur 50603, Malaysia; icmen@icteo.org; 2 Department of Chemistry, Faculty of Science, University of Malaya, Kuala Lumpur 50603, Malaysia; chchuah@um.edu.my; 3 Department of Mechanical Engineering, Southern Taiwan University of Science and Technology, Nan-Tai Street, Yongkang Dist., Tainan City 710, Taiwan; admin@icteo.um.edu.my

**Keywords:** starch, empty fruit bunch, epoxidized palm oil, epoxidized soybean oil, compatibilizer

## Abstract

This study examined the development of starch/oil palm empty fruit bunch-based bioplastic composites reinforced with either epoxidized palm oil (EPO) or epoxidized soybean oil (ESO), at various concentrations, in order to improve the mechanical and water-resistance properties of the bio-composites. The SEM micrographs showed that low content (0.75 wt%) of epoxidized oils (EOs), especially ESO, improved the compatibility of the composites, while high content (3 wt%) of EO induced many voids. The melting temperature of the composites was increased by the incorporation of both EOs. Thermal stability of the bioplastics was increased by the introduction of ESO. Low contents of EO led to a huge enhancement of tensile strength, while higher contents of EO showed a negative effect, due to the phase separation. The tensile strength increased from 0.83 MPa of the control sample to 3.92 and 5.42 MPa for the composites with 1.5 wt% EPO and 0.75 wt% ESO, respectively. EOs reduced the composites’ water uptake and solubility but increased the water vapor permeability. Overall, the reinforcing effect of ESO was better than EPO. These results suggested that both EOs can be utilized as modifiers to prepare starch/empty-fruit-bunch-based bioplastic composites with enhanced properties.

## 1. Introduction

In recent years, the development of bioplastic composites from renewable resources has drawn significant attention due to the growing environmental concerns from petroleum-based plastics and the shortage of petroleum resources [1,2]. Bioplastic composites developed from starch as low-cost and renewable materials have attracted much attention. Starch is one of the most abundant biopolymers in the world, along with chitin and cellulose. Comparatively, starch is the cheapest material for preparing the bioplastics, as compared to other sources, such as protein, chitosan, gelatin, and poly(lactic acid) (PLA) [3,4]. Sugarcane and corn are cultivated for animal feed and food purposes in Malaysia, while the main local sources of starch are cassava [5]. Starch-based bioplastics comprise the largest production capacity (21.3%) in the bioplastic market, while the remaining production is based on PLA, poly(butylene adipate-co-terephthalate) (PBAT), bio-based polyethylene (PE), and others [6]. Pure starch-based bioplastics are very brittle and need the incorporation of plasticizers such as glycerol or polyols to make them easier to handle [7,8]. However, the application of these bioplastics has been hampered because of their great water sensitivity and low mechanical properties [9]. Lignocellulosic fibers have a promising future as the reinforcements of bioplastic composites on account of their high strength, biodegradability, and low cost [10]. Moreover, the fibers are miscible with starch potentially by forming hydrogen bonds. Though fibers could enhance the performance of starch-based bioplastics to a certain extent, both biopolymers and plasticizers are hydrophilic, resulting in a poor water resistance of the composites [11,12].

In order to improve these properties, various strategies have been developed, including blending with other biodegradable polymers (e.g., poly(lactic) acid (PLA), polycaprolactone (PCL), and poly(butylene adipate-co-terephthalate) (PBAT)) or lipids (e.g., palmitic acid, stearic acid, beeswax, and plant oils) [13,14,15]; adding the crosslinking agents (e.g., sodium tri-metaphosphate, epichlorohydrin, glutaraldehyde, and various poly(carboxylic acid)s, such as citric acid and 1,2,3,4-butane tetracarboxylic acid) [16,17,18]; and chemical modification of starch (e.g., esterification with carboxylic acid, acyl chloride and acid anhydride, acetylation with acetic anhydride, and oxidation with hydrogen peroxide) [19].

Epoxidized oils (EOs) represent one of the most commercially important materials because they are cheap and can be produced in large scale [9]. Currently, several studies have been conducted on the incorporation of EOs into biopolymer-based composites, to reduce their moisture sensitivity and enhance their mechanical properties. The addition of EOs could not only provide hydrophobicity to the composites, but also induce possible crosslinking effect with the biopolymers, to form a strong composite network [20,21]. In addition, EOs can act as reactive plasticizers or compatibilizers to polymers, such as PLA, due to the reactivity of epoxy groups [1,22]. However, the studies about the application of EO in the starch-based bioplastics are still rare. Epoxidized soybean oil (ESO) is one of the most widely utilized EOs throughout the world. Recently, few studies have applied it to modify the properties of starch-incorporated or -based composites. For example, Xiong, Yang, Feng, Zhang, Zhang, Tang, and Zhu [22] fabricated a composite based on PLA and maleic anhydride grafted starch by melt-compounding with ESO. Furthermore, relevant studies have used ESO as the modifier for thermoplastic starch, by melt reactive blending. ESO has brought about a partial crosslinking of the epoxide ring with hydroxyl groups of starch. The tensile strength increased by 300% with the addition of 3 wt% ESO [1].

Palm oil is the cheapest and most abundant commodity oil in Malaysia. Developing new products from palm oil, such as epoxidized palm oil (EPO), has attracted considerable interest [23]. EPO has been used as an additive in plastic, a starting material to make polyol, and a pre-polymer in surface coating [24]. ESO has been established as a component in industrial production successfully, while EPO has just been developed as a potential modifier recently. Oxirane oxygen contents of EOs determine the number of reaction points and are suggested to be the key factor which affects the reaction efficiency of EOs and their performance in the composites [25,26]. ESO shows higher oxirane oxygen percent and, thus, a greater possibility of reactions, as compared to EPO [27]. Therefore, it is meaningful to compare the effect of EPO on the properties of biopolymer-based bioplastic composites with ESO and represent EPO as an alternative constituent for bioplastic composites.

In palm oil plant, after the palm fruits have been detached for palm oil extraction, the rest is called oil-palm empty-fruit-bunch (EFB) fibers [28]. EFB fibers are the most abundant wastes in Malaysia. They are a form of fibrous lignocellulosic residues and composed of cellulose, hemicellulose, and lignin. EFB fibers have a considerable potential as reinforcing fibers [29]. Therefore, to create a new market for the palm oil industry, it attracts great interest to investigate the effect of EPO on the properties of starch/EFB-based bioplastics.

Only few a research studies have been carried out on biopolymers, fillers, and oil modifiers blended composites. Tanrattanakul and Saithai [27] prepared bioplastic-organoclay nanocomposites with ESO of different epoxide contents. Balart et al. [30] processed the composites with PLA and hazelnut-shell flour plasticized by epoxidized linseed oil. Meng et al. [31] compounded cellulose nanofibrils and ESO into PLA, to achieve high strength and toughness. In general terms, most of the studies were linked to PLA-based bio-composites. The interactions of EO and biopolymers/fillers led to the enhanced properties of the composites. However, as far as we know, no former study has proposed EO to modify starch/EFB-based composites.

In the present study, starch/EFB-based bioplastic composites were reinforced with EO by emulsion casting and compression molding. The interactions among components and interfacial adhesion within the composites were investigated by FTIR and SEM. Furthermore, tensile test, water resistance, and thermal analysis were utilized, to demonstrate the influence of EO on the physical and thermal properties of the composites. This study sheds light on the choice of EO with different epoxy values, to improve the properties of the bioplastic composites.

## 2. Materials and Methods

### 2.1. Materials

Oil-palm empty-fruit-bunch (EFB) fibers (moisture content 9.40%, powder) were provided by LGC Scientific SDN BHD located in Selangor (Malaysia). The EFB fibers were subjected to simple treatment before use. The same weights of EFB fibers and NaOH solution (10 wt%) were blended and heated at 180 °C, for 30 min, in the oven. Afterwards, the obtained specimens were washed and dried. The particles were ground and sieved, using 63 μm mesh size. The chemical composition of the resulting fibers was as follows: 44.70% cellulose, 24.55% hemicellulose, and 15.45% lignin. The other properties of the fibers are not shown here.

Native cassava starch (moisture content 11.43%, amylose content around 21.2%), commercial refined palm oil (PO, minimum purity 99%), and commercial soybean oil (SO, minimum purity 99%) were supplied by LGC Scientific SDN BHD. Acetic acid (CH_3_COOH), anhydrous calcium chloride (CaCl_2_), anhydrous sodium sulfate (Na_2_SO_4_), glycerol, hydrogen peroxide (30% H_2_O_2_), sodium carbonate (Na_2_CO_3_), sodium chloride (NaCl), sodium hydroxide (NaOH), sulfate acid (H_2_SO_4_), and Tween 80 were purchased from Friendemann Schmidt Chemicals (Parkwood, Australia) and used as received. The chemical reagents were analytical grade.

### 2.2. Methods

#### 2.2.1. Epoxidation of Palm Oil (PO) and Soybean Oil (SO)

EPO and ESO were synthesized by slightly modifying the procedures of Kim and Sharma [26]. A 500 mL four-neck reactor, equipped with a mechanical stirrer, a thermometer, a dropping funnel, an oil bath, and a cold-water condenser, was used. For the epoxidation of PO, 200 g PO, 22.26 g CH_3_COOH, and a required amount of H_2_SO_4_ (2% of the H_2_O_2_-CH_3_COOH mixture) were blended and stirred for 30 min. For the epoxidation of SO, the mixture of 200 g SO, 50.40 g CH_3_COOH, and H_2_SO_4_ (2% of the H_2_O_2_-CH_3_COOH mixture) were prepared similarly. Afterwards, 84.09 g 30% H_2_O_2_ (158.80 g for SO) was added at 2 mL/min, keeping the reaction at 60 °C for 8 h. Next, the solution was washed twice with 50 °C Na_2_CO_3_ solution (5 wt%), followed by 50 °C distilled water. The oil layer was separated by decantation and dried overnight, at 60 °C, with Na_2_SO_4_. Na_2_SO_4_ was removed by filtration with Whatman No. 4 filter paper. The oxirane oxygen contents of EPO and ESO were 2.95% and 6.23%, respectively, by following the AOCS Official Method Cd 9-57.

#### 2.2.2. Preparation of Starch/Empty Fruit Bunch-Based Bioplastic Composites

Firstly, starch, glycerol, and EFB fibers (70/30/5, wt%) were dispersed in the distilled water and stirred continuously, at 90 °C, for 30 min. Then, different contents of EPO or ESO (0.375, 0.75, 1.5, and 3 wt% of starch and glycerol) were added with Tween 80 (25 wt% of EO) as the emulsifier. The solution was homogenized at 20,000 rpm, for 2 min, using a rotor-stator homogenizer. Afterwards, the emulsion with 2.00 g solid was spread evenly on the glass plates (Diameter 15 mm) and dried under 40 °C, in an oven, for 48 h.

The emulsified films showed weak strength, and it was too difficult to detach them off the plates intact. Moreover, the condensation reaction between EO and starch requires high temperature. Thus, the films (70 g) were further processed by a Hydraulic Molding Test Press (GT-7014-A30C), at 130 °C and 13.79 Mpa, for 6 min, with an aluminum mold of 200 mm × 200 mm × 1 mm. The fabricated specimens were maintained at 53% RH (relative humidity) for at least two days before further analysis. The formulation for each sample is depicted in Table 1.

### 2.3. Fourier Transform Infrared Spectroscopy (FTIR)

FTIR measurements of all the composites were recorded by Spectrum 400 FTIR/FT-FIR spectrometer. The samples were scanned from 4000 to 450 cm^−1^, at a 4 cm^−1^ resolution and an accumulation of 32 scans. The samples were fabricated by the KBr-disk method.

### 2.4. Scanning Electron Microscopy (SEM)

The surface morphologies of the composites were performed by SEM (Phenom Pro X), at an accelerated voltage of 15 kV. The surfaces were observed after a gold sputter coating with Polaron SC7640.

### 2.5. Diffraction Scanning Calorimetry (DSC)

The melting temperatures of the composites were determined by a DSC Q20 (TA Instrument). The thermograms were analyzed from −50 to 250 °C, with a nitrogen flow of 20 mL/min and a heating rate of 10 °C/min.

### 2.6. Thermogravimetric Analysis (TGA)

TGA was carried out by a PerkinElmer TGA 4000, from 30 to 600 °C, under a nitrogen flow (10 mL/min) and a heating rate of 10 °C/min. The mass loss (TG) and derivative thermogravimetric curves (DTG) were collected.

### 2.7. Tensile Properties

A tensile test was carried out, conforming to ASTM D638, by means of an AGS-X universal/tensile tester (Shimadzu, Japan), at a crosshead speed of 5 mm/min. The bioplastics were cut to dimensions of a dumbbell shape (6.4 cm (length) × 1.0 cm (width)). Mechanical properties for each bioplastic were obtained by averaging 5 experimental values.

### 2.8. Water Vapor Permeability (WVP)

To measure the WVP of the composites, the cup was filled with 1.0000 g CaCl_2_ and covered with the circle sample. Then the cup was sealed and placed in a desiccator containing saturated NaCl solution, at 25 °C. While 0% RH was maintained inside the test cup by CaCl_2_, the saturated NaCl solution provided 75% RH. Weight changes of the cup were recorded at 24 h intervals, for a 7-day period, and plotted as a function of time. WVP (g m/m^2^ h Pa) for each specimen was determined according to the following equations:

(1) $$WVTR\;=\;\frac{w}{t\times A}$$

(2) $$WVP\;=\;\frac{WVTR\;\times X}{P\;\times \;\left(R_{1}-\;R_{2}\right)}$$

where *w*/*t* represents the weight increase per elapsed unit time; *A* means the exposed surface area of the composites; *X* represents the average thickness of the composites (1.40–1.66 mm), which was determined by using a hand-held digital micrometer (Mitutoyo RQU342, Japan) with a precision of 0.01 mm; *P* indicates the saturation water vapor pressure between two sides of composites (31.7 mbar at 25 °C); *R*_1_ presents the RH inside the desiccator; and *R*_2_ is the RH in the cup.

### 2.9. Water Uptake and Solubility

The sample with dimensions of 10 mm × 15 mm was dried and submitted to a desiccator with a controlled 75% RH for 3 days. The water uptake was expressed as absorbed water (g) per dry matter (g). Regarding water solubility, the sample with the above dimensions was dried and placed in the cup with 50 mL distilled water, for 24 h, at room temperature. The insoluble pieces were collected and dried at 105 °C, in an oven, for 24 h. Water solubility was calculated from weight loss between the initial and final dry weight of the composites. Three measurements were carried out for each specimen.

### 2.10. Statistical Analysis

Data were presented as means ± standard deviation and analyzed through variance analysis (ANOVA) and Turkey’s multiple range tests, using SPSS 19.0 (SPSS Inc., Chicago, IL, USA). A probability value of *p* < 0.05 was considered as statistical differences.

## 3. Results and Discussion

### 3.1. FTIR

**Figure 1:**
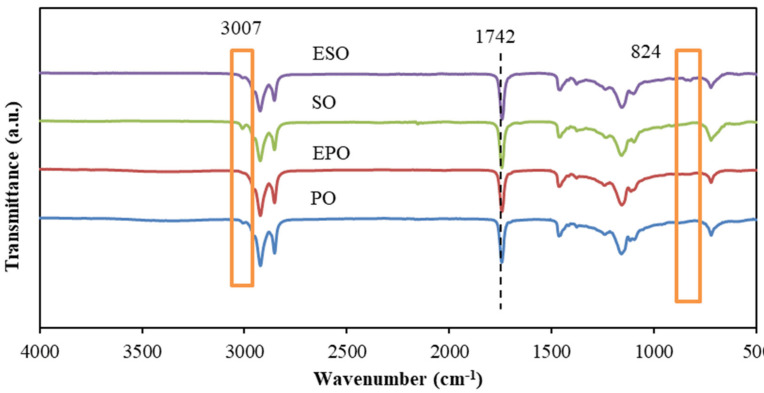
FTIR spectra of raw oils and epoxidized oils.

The FTIR spectra for raw oils and epoxidized oils are presented in Figure 1. The general spectra of PO and SO were similar. The bands displayed at 3007 cm^−1^ are associated with C-H stretching vibrations of C=C-H [27]. The terminal methyl groups of the triglyceride present strong C-H stretching vibrations in the 2922 cm^−1^, while the stretching vibrations of methylene groups in C-H show the bands at 2853 cm^−1^. Two strong characteristic ester groups arising from C=O and C-O stretching vibrations are situated at 1742 and 1236 cm^−1^, respectively [24]. In the spectrum of PO, the band intensity for alkene group in the 3007 cm^−1^ regions was lower, as compared to SO, indicating that there were more double bonds in SO [32].

Regarding EPO and ESO, the epoxidation reaction was apparent by observing the decreased intensity of the bands for the double bonds (3007 cm^−1^), and the appearance of bands at 824 cm^−1^ attributed to the epoxide groups (C-O-C) [1]. The remaining signals for double bonds revealed incomplete epoxidation conversion. The intensity of the bands for ESO at 824 cm^−1^ were higher compared to that of EPO, which demonstrated higher degree of epoxidation of ESO. This was also confirmed by the oxirane oxygen contents of EPO and ESO, which were 2.95% and 6.23%, respectively. Therefore, it was expected that ESO had a better chemical reactivity. 

**Figure 2:**
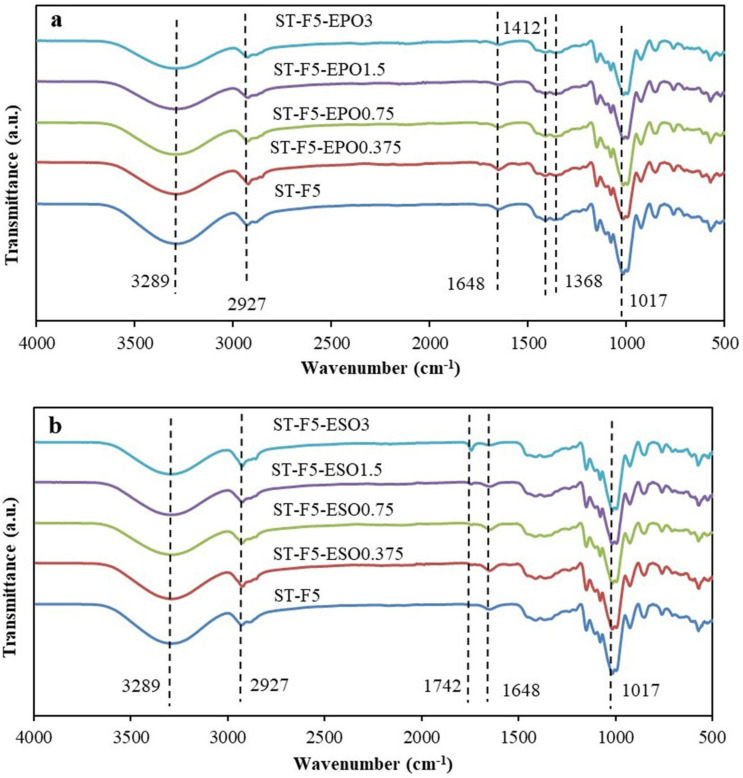
FTIR spectra of (**a**) bioplastic composites with EPO and (**b**) bioplastic composites with ESO.

The FTIR spectra of the composites with EO did not show much change due to the similar peak characteristic of EPO and ESO and their low loading levels (Figure 2a,b). The bands around 3289–3303 cm^−1^ belong to stretching vibrations of O-H [33]. The peaks at 2922–2928 cm^−1^ are assigned to the C-H stretching vibrations. The typical peaks for C=O of plant oils at 1742 cm^−1^ were clearly detected in the bioplastics with ESO contents increasing. The bands around 1646–1652 cm^−1^ are characteristic of the water molecules in the starch [33]. The bands at 1411–1417 cm^−1^ and 1356–1368 cm^−1^ are associated with bending and deformation of C-H, respectively. The strong bands in the 1016–1019 cm^−1^ regions present O-C stretching vibrations in the anhydroglucose ring [33].

It was found that the peaks for O-H stretching shifted to higher wavenumbers after the introduction of EO. These shifts could reveal the interactions between the oxirane groups from EO and the hydroxyl groups of starch and fibers [27]. Belhassen, Vilaseca, Mutjé, and Boufi [1] reported that oxirane groups of ESO were supposed to crosslink with the hydroxyl groups of starch. Moreover, partial oxirane moieties might be converted into hydroxyl groups by the ring opening reaction with H_2_O during the drying process of the casting solutions [27]. The resulted oil-based bio-polyols would facilitate the interactions among the composites due to their hydroxyl groups. Given the low content of EO and the interference of the bands of starch, the condensation reaction between EO and starch was not detected.

### 3.2. SEM

**Figure 3:**
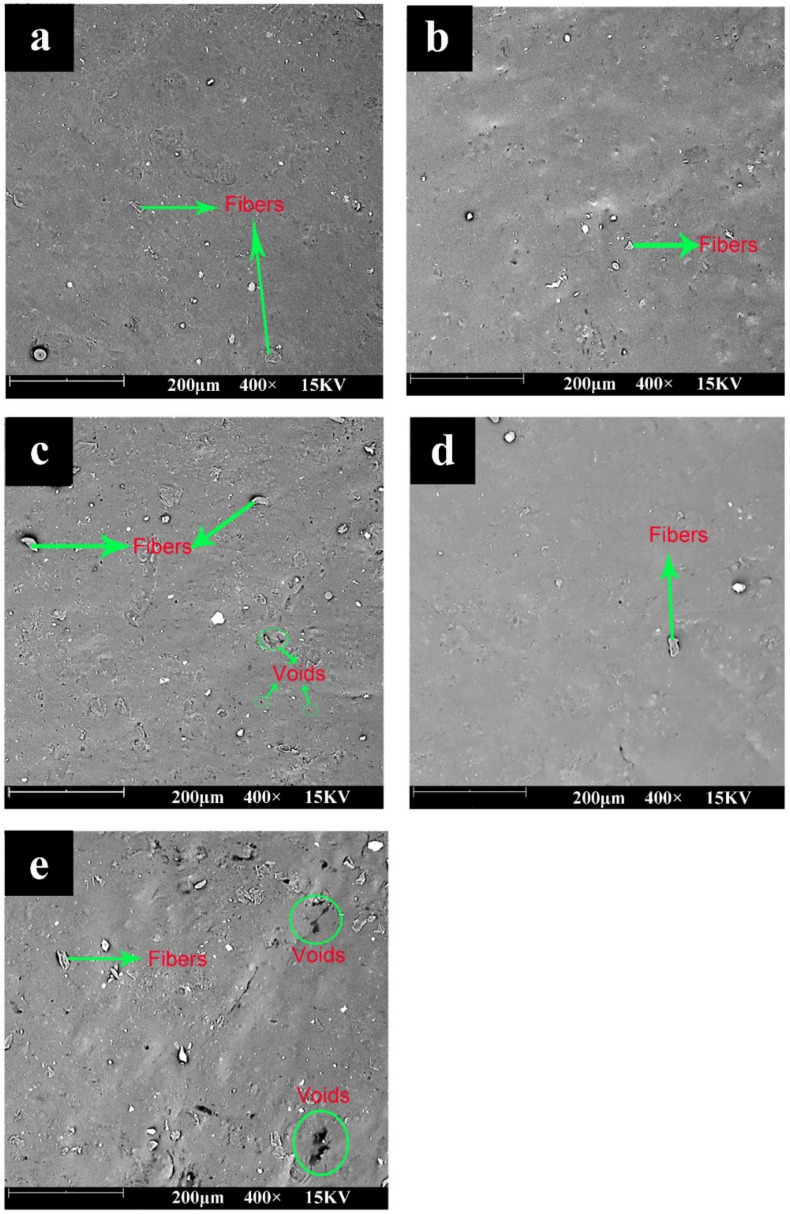
SEM photographs of prepared bioplastic composites: (**a**) ST-F5, (**b**) ST-F5-EPO0.75, (**c**) ST-F5-EPO3, (**d**) ST-F5-ESO0.75, and (**e**) ST-F5-ESO3.

The oil distribution in the emulsified bio-composites depends on the composition of oils, interactions between oils and biopolymers, and structural development during the course of drying [14,34]. The surfaces of the prepared bioplastic composites are shown in Figure 3. The surface of ST-F5 was smooth, with its fibers dispersed uniformly. Because of the considerable particle size of EFB fibers, the interfacial adhesion between starch and EFB fibers was compromised.

Both the types and contents of EO had significant effects on the surface morphology of the composites. When 0.75 wt% EO was blended, smoother and more compact surfaces were observed, indicating the improved compatibility of EFB fibers and starch. ESO gave rise to smoother a surface, in comparison with EPO. The epoxide group can establish physical or chemical interactions with the hydroxyl groups in starch and fibers, at high temperature, during compression molding [1,30]. The higher oxirane oxygen content of ESO enabled more reaction points with starch through crosslinking, as compared to EPO [26]. Thus, ESO provided a stronger effect on starch and EFB fillers, resulting in a better biopolymer-particle adhesion.

Incorporating high concentrations of EO (3 wt%) generated remarkable changes of the composites’ surface structure. Many cavities corresponding to the oil-rich phase were observed, along with discontinuities, reflecting the poor compatibility of the components. Moreover, the composites with ESO showed more voids, when compared to these with EPO. Extra EO would aggregate and migrate due to the phase separation during drying steps of films [35]. The interactions among EO, starch, and fibers were insufficient to prevent phase separation during the solution evaporation [36]. This was consistent with the poor mechanical properties, as discussed below. Thus, 0.75 wt% EO was considered to be a good compatibilizer level for the composites.

### 3.3. DSC

**Figure 4:**
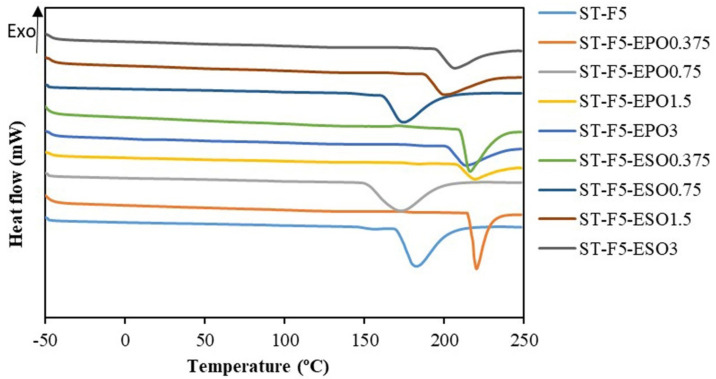
Diffraction scanning calorimetry (DSC) curves of all the bioplastic composites.

DSC thermograms and thermal parameters of starch-based bioplastic composites are displayed in Figure 4 and Table 2, respectively. On the DSC thermogram of ST-F5, the broad endothermic peak is attributed to the melting peak. Their T_onset_ (initial melting temperature) and T_m_ (melting temperature) were 171.0 and 182.3 °C, respectively. Comparatively, the composites with EPO showed higher T_m_ and T_onset_ compared to those with ESO at the same EO concentration, except 0.75 wt%. The T_m_ and T_onset_ of the composites were increased significantly after the incorporation of 0.375 wt% EPO or ESO. These results suggested lower molecular mobility of starch chains, due to the strengthened interaction between starch and EO, which was also confirmed by the huge increase of tensile strength, as shown below. However, 0.75 wt% EO reduced the T_m_ and T_onset_ of the composites remarkably. A possible explanation of the reduced T_m_ was that EOs were slightly excessive and cannot crosslink or interact sufficiently with starch/EFB, considering the fact that the tensile strength did not show a double increase, as compared to the composites with 0.375 wt% EO. Moreover, the presence of few voids in the SEM images of the composites with 0.75 wt% verified the outcome. Therefore, excess EO might occupy intermolecular spaces among starch like glycerol and facilitate the reduction of hydrogen bonds of starch, thus increasing the mobility of biopolymer chains [37]. The dangling chains of unreacted EO in the composite would increase the free volume, subsequently lowering the T_m_. Moreover, it was reported that T_m_ shifted to a lower temperature due to the plasticization effect [32,36]. However, 1.5–3 wt% EO might deteriorate the phenomenon, possibly due to the incompatibility of excess EO and starch/EFB, thus hampering the melting of the composites. This was consistent with the behavior of phase separation, as shown by the SEM.

### 3.4. TGA

**Figure 5:**
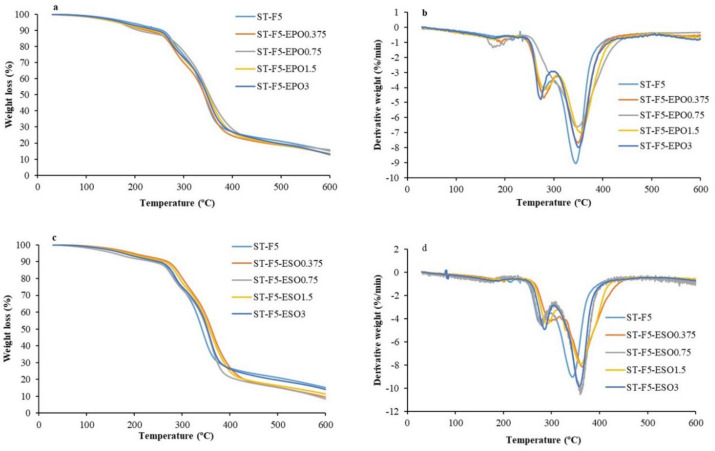
Thermal degradation curves of all the bioplastics. (**a**) TG curves of the bioplastic composites with EPO, (**b**) DTG curves of the bioplastic composites with EPO, (**c**) TG curves of bioplastic composites with ESO, and (**d**) DTG curves of the bioplastic composites with ESO.

The thermogravimetric (TG) and derivative thermogravimetric (DTG) curves for the bioplastics with types of EOs are shown in Figure 5a–d. The thermal degradation behavior of the composites incorporated with EO was similar to the control samples. The weight loss of the bioplastics primarily occurred in three steps. In the initial step, the weight loss of 10–20% was found from 30 to 270 °C, which is related to the loss of water and glycerol. In the second stage of 270–420 °C, the weight loss of the bioplastics corresponds to the degradation of glycerol, starch, EO, and fibers. Finally, in the range of 420–600 °C, the degradation is mainly attributed to carbonaceous residues. T_5%WL_ of the composites was reduced by the incorporation of EO except 0.375 wt% ESO (Table 3). 

The DTG curves showed that two maximum decomposition rate peaks were observed. T_max1_ and T_max2_ of ST-F5 occurred at 276.0 and 346.6 °C, respectively. No obvious difference of T_max1_ and T_max2_ was observed between the composites with and without EPO. However, the addition of ESO improved T_max1_ and T_max2_ of the bioplastics, which was in the range of 279.6–298.5 °C and 357.7–364.3 °C, respectively. This revealed that the thermal stability of the bioplastics was increased by the introduction of ESO, which can be ascribed to the stronger interaction of ESO with fibers and starch.

### 3.5. Mechanical Properties

**Figure 6:**
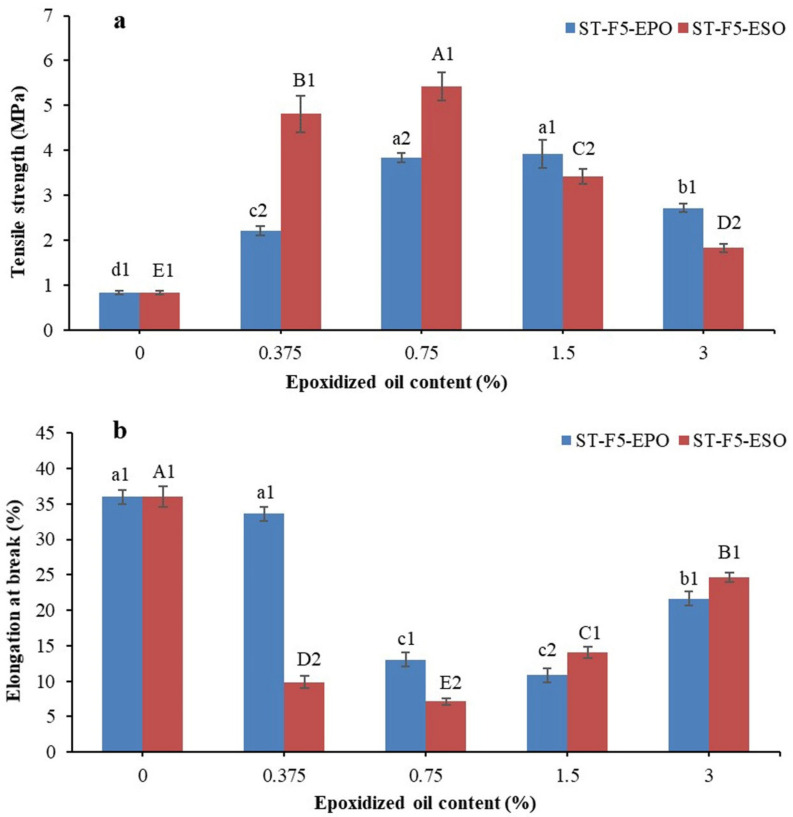
Tensile strength (**a**) and elongation at break (**b**) of the bioplastic composites. In regard to a–d and A–E, different letters within the same indicator indicate significant difference among samples (*p* < 0.05). (1 and 2) Different numbers indicate significant difference between the formulations with palm oil (PO) and EPO at the same concentration (*p* < 0.05).

The impact of various EOs on the tensile properties of the bioplastic composites is described in Figure 6a,b. The composites without EOs were fairly flexible materials with an elongation at break of 36.01% and a tensile strength of 0.83 MPa. The addition of 0.375–0.75 wt% EO induced a huge increase of the tensile strength (*p* < 0.05). The tensile strength reached a maximum of 3.92 MPa with 1.5 wt% EPO and 5.42 MPa with 0.75 wt% ESO. The major reason for the increase of tensile strength was likely to be the consequence of the strong interactions between EO and starch/EFB. It was reported that EO could accumulate on the fiber surfaces by physical or chemical interactions. These interactions allowed load transfer from EFB fibers to starch, which subsequently enhanced the reinforcing ability [26]. The strengthened interaction led to a more brittle fracture, as revealed by a downward elongation at break (*p* < 0.05). With the EO contents increasing from 1.5 to 3 wt%, the composites revealed the higher elongation at break and lower tensile strength. Similar results have been observed in starch-based films incorporated with various plant oils [20,38]. The excess EO could not interact or crosslink sufficiently with starch/EFB and might contribute to the plasticization of the starch, like the non-epoxidized oils [20,36]. The decrease of tensile strength reflected the reduced interaction among starch molecules and might be caused by the formation of EO–EO interaction at higher oil contents, due to their flexible properties. As a result, the looser structure of the composites was developed. This corresponded to the microvoids of the EO-rich phase, as observed by the SEM micrographs. 

Comparatively, ESO exhibited higher reinforcing effect on the composites than EPO (*p* < 0.05) due to higher oxirane oxygen content [30]. However, bioplastic composites reinforced with ESO were too brittle and would crack when cut after conditioned. Belhassen, Vilaseca, Mutjé, and Boufi [1] also came to the same conclusion and suggested increasing the quantity of EO or using an EO with lower reactivity. Considering the poor compatibility between starch and high contents of EO, EPO was a suitable choice to alleviate the rigidification effect of ESO.

In conclusion, the results highlighted that the effect of EOs on the composites’ tensile properties was determined by the types and concentrations. Low contents of EOs primarily acted as compatibilizers, to improve the tensile strength. The composites became very brittle after the addition of ESO and would crack when they were cut into small pieces. However, extra EOs (3%) behaved mainly as low-efficient plasticizers to soften the composites.

### 3.6. Water Uptake and Solubility

**Figure 7:**
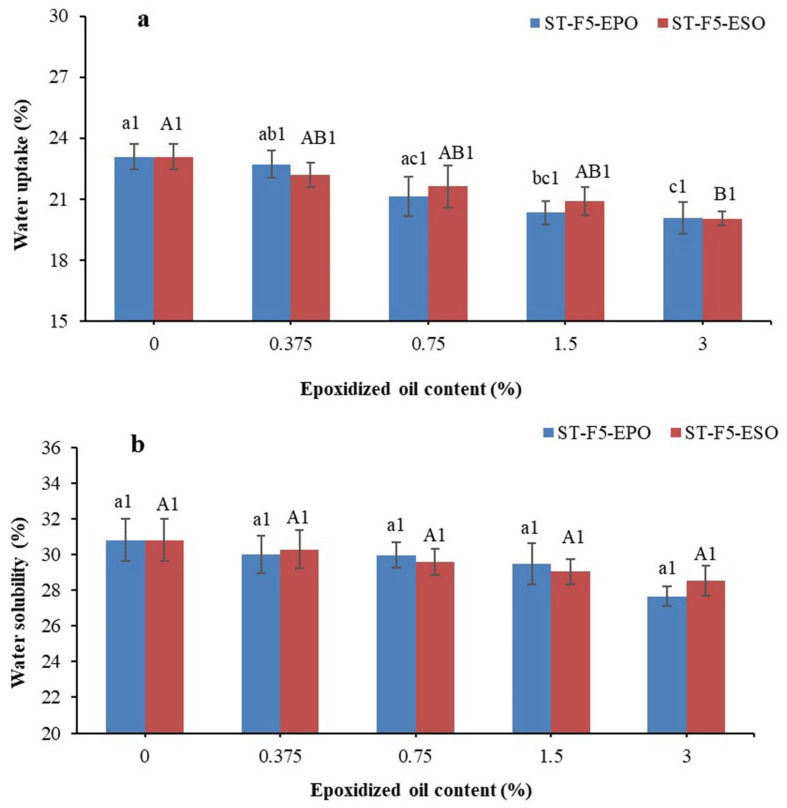
(**a**) Water uptake and (**b**) solubility of the bioplastic composites. In regard to a–c and A,B, different letters within the same indicator indicate significant difference among samples (*p* < 0.05). (1) Same number indicates no significant difference between the formulations with PO and EPO at the same concentration (*p* > 0.05).

The water sensitivity is dependent on both the composition of the composites and the interactions among the components [39,40,41,42]. Water uptake and solubility of all the composites are investigated in Figure 7a,b. The results revealed that water uptake of the composites exhibited lower values owing to the incorporation of EO (*p* < 0.05), while the solubility of the composites slightly decreased with the concentrations of EO increasing (*p* > 0.05). There was no obvious difference of water uptake and solubility between the composites with EPO and ESO (*p* > 0.05).

The long fatty acid chains of EO can protect the hydroxyl groups of starch/EFB from moisture absorption and swelling due to their hydrophobicity [43]. This might be also explained by the enhanced interactions among EO, starch, and fibers, decreasing the availability of hydroxyl groups [21,35]. The effect of both EO on water uptake and solubility did not show huge reduction. This confirmed the insufficient reactivity between EO and starch/EFB, which cannot resist the water molecules greatly [22].

### 3.7. WVP

**Figure 8:**
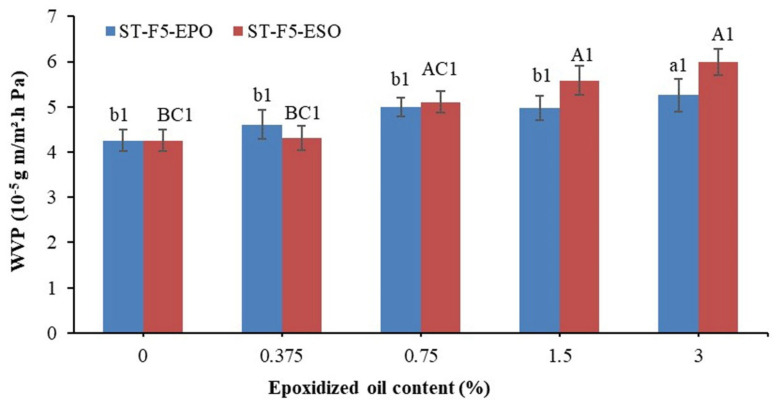
Water vapor permeability (WVP) of the bioplastic composites. In regard to a,b and A–C, different letters within the same indicator indicate significant difference among samples (*p* < 0.05). (1) Same number indicates no significant difference between the formulations with PO and EPO at the same concentration (*p* > 0.05).

As is well-known, the addition of lipids is one of two primary means to reduce the WVP, along with the addition of crosslinking agents [14,44]. Many factors, such as the composition and amount of oils added, interactions among components, drying process, and the final microstructure, affect the WVP of the composites [39,45]. Moreover, the addition of plant oils could increase the tortuosity of water transfer in the composites and contribute to the water barrier of the composites [20,46]. The WVP of all the composites is shown in Figure 8. In this study, the permeability value of ST-F5 was 4.25 × 10^−5^ g m/m^2^ h Pa. However, the WVP increased gradually as EOs were incorporated. The similar behavior was also found in several studies [36,47]. The WVP of the composites with EPO and ESO showed no significant difference (*p* > 0.05).

Firstly, this might be related with the number of microvoids on the surface of the composites which would facilitate the water transfer [48,49,50], as verified by the SEM graphs. Next, the interactions between lipid phase and biopolymer chains in o/w emulsion films might just increase the external tortuousness and take negligible effect on the moisture barrier of emulsified films [51,52,53,54]. In the end, EO and Tween 80 might increase the free volume among starch chains due to their plasticization effect, which facilitated the water transfer [55,56]. Overall, it can be concluded that the WVP would not be reduced by simply adding EO to the composites.

## 4. Conclusions

In this work, starch/EFB-based bioplastic composites were modified by EPO or ESO, at various levels. Incorporating EOs caused the presence of intense interactions between the epoxy groups from EOs and the hydroxyl groups from starch or fibers. Low contents of EO, especially ESO, resulted in a smoother surface of the composites, while high contents of EO induced many voids and discontinuities because of phase separation. The T_m_ of the composites was increased by the incorporation of EOs. The thermal stability of the bioplastics was increased by the introduction of ESO, which can be ascribed to the strong interaction of ESO with starch/EFB. Consequently, a small amount of EO behaved as the compatibilizer and promoted the mechanical properties of the bioplastic composites sharply, while higher contents of EO led to a negative effect, due to the phase separation. Moreover, the water uptake and solubility declined moderately while the WVP increased after the introduction of EO. Overall, the reinforcing effect of ESO at the proper amount was better than EPO, but the composites with ESO were too brittle to cut in designed condition. The addition of EO is a good strategy to develop the bioplastic composites with favorable performance.

## Figures and Tables

**Table 1:** Formulations for starch/empty fruit bunch (EFB)-based bioplastic composites.

Sample Names	Starch (wt%)	Glycerol (wt%)	EFB Fibers (wt%)	EPO (wt%)	ESO (wt%)	Tween 80 (wt%)
ST-F5	70	30	5	-	-	-
ST-F5-EPO0.375	70	30	5	0.375	-	0.09375
ST-F5-EPO0.75	70	30	5	0.75	-	0.1875
ST-F5-EPO1.5	70	30	5	1.5	-	0.375
ST-F5-EPO3	70	30	5	3	-	0.75
ST-F5-ESO0.375	70	30	5	-	0.375	0.09375
ST-F5-ESO0.75	70	30	5	-	0.75	0.1875
ST-F5-ESO1.5	70	30	5	-	1.5	0.375
ST-F5-ESO3	70	30	5	-	3	0.75

Notes: ST and F indicate starch and EFB fibers, respectively. The number in each code represents its weight percentage in the composites. EPO, epoxidized palm oil; ESO, epoxidized soybean oil.

**Table 2:** Melting temperature of the prepared bioplastic composites.

Formulations	T_onset_ (°C)	T_m_ (°C)
ST-F5	171.0	182.3
ST-F5-EPO0.375	215.4	220.1
ST-F5-EPO0.75	152.2	172.9
ST-F5-EPO1.5	208.8	219.1
ST-F5-EPO3	202.6	213.7
ST-F5-ESO0.375	210.5	216.3
ST-F5-ESO0.75	162.6	174.7
ST-F5-ESO1.5	188.6	199.7
ST-F5-ESO3	195.4	206.8

**Table 3:** Thermal degradation parameters of the prepared bioplastic composites.

Formulations	T_5%WL_ (°C)	T_max1_ (°C)	T_max2_ (°C)
ST-F5	189.4	276.0	346.6
ST-F5-EPO0.375	166.3	279.5	349.4
ST-F5-EPO0.75	168.7	-	345.8
ST-F5-EPO1.5	167.8	284.1	354.1
ST-F5-EPO3	175.8	273.1	350.1
ST-F5-ESO0.375	200.4	298.5	364.3
ST-F5-ESO0.75	159.6	279.6	359.5
ST-F5-ESO1.5	188.3	290.3	359.6
ST-F5-ESO3	179.4	285.2	357.7

T_5%WL_: temperature at 5% weight loss. T_max_: temperature at maximum weight loss rate.
